# Comprehensive multi-omics analysis identifies NUSAP1 as a potential prognostic and immunotherapeutic marker for lung adenocarcinoma

**DOI:** 10.7150/ijms.102331

**Published:** 2025-01-01

**Authors:** Jun Ma, Wenjing Zhu, Yuan Wang, Hongmei Du, Ling Ma, Lisha Liu, Chao Niu, Songlei Li, Kai Zhang, Enwu Yuan

**Affiliations:** 1 Department of Laboratory Medicine, Third Affiliated Hospital of Zhengzhou University, Zhengzhou, Henan, China.; 2 Zhengzhou Key Laboratory for In vitro Diagnosis of Hypertensive Disorders of Pregnancy, Zhengzhou, Henan, China.

**Keywords:** NUSAP1, LUAD, immunotherapy, m6A, scRNA-seq

## Abstract

While NUSAP1's association with various tumors is established, its predictive value for prognosis and immunotherapy in lung adenocarcinoma (LUAD) remains unconfirmed. We analyzed Nucleolar Spindle-Associated Protein 1 (NUSAP1) gene expression in TCGA and GTEx datasets and validated it in clinicopathological tissues using qRT-PCR and immunohistochemistry. Additionally, we investigated NUSAP1's relationship with patient prognosis across TCGA and five GEO cohorts. The IMvigor210 cohort was utilized to explore NUSAP1's association with immunotherapy efficacy. Furthermore, single-cell RNA-sequencing data was used to examine the correlation between NUSAP1 and immune cell infiltration. Finally, we analyzed the relationship between NUSAP1 and m6A methylation. NUSAP1 expression was significantly elevated in tumor tissues, correlating with poorer prognosis in LUAD patients. It exhibited a significant correlation with immune cell infiltration in the tumor microenvironment, predominantly expressed in Tprolif cells. LUAD patients with heightened NUSAP1 expression may derive greater benefit from anti-PD-L1 treatment. Additionally, NUSAP1 was tightly linked with m6A methylation. Enrichment analysis revealed its association with key biological functions, including lipid metabolism and cell cycle regulation. Our comprehensive analysis underscores NUSAP1's potential as a prognostic and immunotherapeutic biomarker for LUAD, warranting further investigation.

## Introduction

Nucleolar spindle-associated protein 1 (NUSAP1) serves as a key cell cycle regulatory protein, primarily engaged in mitotic progression, spindle formation, and stabilization 1. Dysregulation of NUSAP1 results in spindle structural abnormalities, culminating in chromosome misaggregation and aberrant cell division, thereby contributing to tumorigenesis 2. Extensive evidence suggests NUSAP1's involvement in the onset and progression of various cancers 3. NUSAP1 plays a pivotal role in driving the progression of pancreatic ductal adenocarcinoma by facilitating epithelial-mesenchymal transition and AMPK phosphorylation 4. Moreover, it serves as a shared genetic signature linking chronic HBV infection to HBV-associated HCC, while also contributing to cisplatin resistance in hepatocellular carcinoma 5, 6. NUSAP1 profoundly influences the proliferation and invasive potential of basal cell carcinoma 7, glioblastoma 8, ovarian cancer 9, as well as breast 10 and nephroblastoma cells 11. Regrettably, there is currently no documented research exploring the correlation between NUSAP1 and the response to immunotherapy in LUAD, highlighting the need for further investigation in this area.

In this study, we delved into the expression profiles and potential prognostic significance of NUSAP1 across multiple publicly available datasets. Furthermore, we meticulously validated these findings in clinical samples using qRT-PCR and immunohistochemistry (IHC). Additionally, we assessed the predictive value of NUSAP1 expression for immunotherapy response in LUAD patients. Lastly, our study explored the correlation between NUSAP1 expression and immune infiltration as well as m6A-related genes. These discoveries offer valuable insights into the potential relevance of NUSAP1 in LUAD.

## Methods

### Data collection

For the TCGA-LUAD and GTEx datasets, RNA sequencing data and related clinicopathologic details were acquired from the UCSC Xena database (https://xenabrowser.net/). Transcriptome data underwent normalization using the log2 (FPKM+1) transformation, and batch effects were adjusted using Combat from the R package "SVA". Normalized expression data from the GSE10072, GSE31210, GSE37745, GSE50081, GSE68465, and GSE42127 cohorts were obtained from the Gene Expression Omnibus (GEO, https://www.ncbi.nlm.nih.gov/geo) database. Data pertaining to the cohort receiving anti-PD-L1 immunotherapy were retrieved by downloading and installing the “IMvigor210CoreBiologies” package from http://research-pub.gene.com/.

### Quantitative reverse-transcription polymerase chain reaction (qRT-PCR)

Following established protocols 12, NUSAP1 mRNA expression was evaluated via qRT-PCR in tumor and paracancerous tissues from 30 LUAD patients who did not receive any radiotherapy or chemotherapy. The following primer-synthesized NUSAP1 sequences were utilized: forward 5ʹ-AGCCCATCAATAAGGGAGGG-3ʹ, reverse 5ʹ-ACCTGACACCCGTTTTAGCTG-3ʹ. Ultimately, the relative expression of NUSAP1 mRNA was determined using the 2-ΔΔCT method.

### Human tissue samples

Paraffin-embedded human lung adenocarcinoma tissues were obtained from The First Affiliated Hospital of Zhengzhou University. The specimens were collected under an Institutional Review Board (IRB) approved protocol (2019-KY-258) with obtained informed consent.

### Immunohistochemistry (IHC) assay

Independent IHC tests were conducted on paraffin-embedded tissues using anti-NUSAP1 (Invitrogen, US). Images were captured using a Leica DM 2500 microscope following the application of secondary antibodies to the slides. Immunostaining intensity was evaluated and scored by two independent observers. A final score, distinguishing between negative (≤4) and positive (>4) NUSAP1 protein expression, was derived by summing the extent of expression and intensity scores.

### Immune cell infiltration analysis

Various databases including the ESTIMATE 13, CIBERSORT 14, xCELL 15, TIMER databases 16, and single-sample genome enrichment analysis (ssGSEA) algorithm were employed to assess the infiltration of different immune cell types in the tumor microenvironment (TME). Furthermore, we scrutinized the expression profile of NUSAP1 across various cell types within the TME using the TISCH database (http://tisch.comp-genomics.org/) 17, including GSE139555, GSE148071, GSE162498, and GSE99254 cohorts.

### Prediction of potential tumor-sensitive drugs targeting NUSAP1

The CellMiner database 18 was utilized to explore potential tumor-sensitive drugs targeting the NUSAP1 gene. Screening criteria included an adjusted P value of less than one in a thousand and a Pearson correlation coefficient exceeding four-tenths. Differences in half-maximal inhibitor dose (IC50) of drugs across different NUSAP1 gene expression levels were analyzed to demonstrate therapeutic sensitivity.

### The pathway richness analysis

Gene Set Variation Analysis (GSVA) and Gene Set Enrichment Analysis (GSEA) were employed using the R packages "GSVA" and "ClusterProfiler", respectively, to evaluate differences in biological processes between NUSAP1-high and NUSAP1-low expression subgroups.

### Statistical analysis

All statistical analyses were conducted using R-4.2.1. Hazard ratios (HRs) and associated 95% confidence intervals were calculated using univariate survival analysis. Kaplan-Meier analysis categorized patients based on high or low NUSAP1 expression levels to assess patient survival. Statistical significance was attributed to changes with a p-value less than 0.05, and all tests were two-sided.

## Results

### High elevated NUSAP1 expression in LUAD

**Figure 1:**
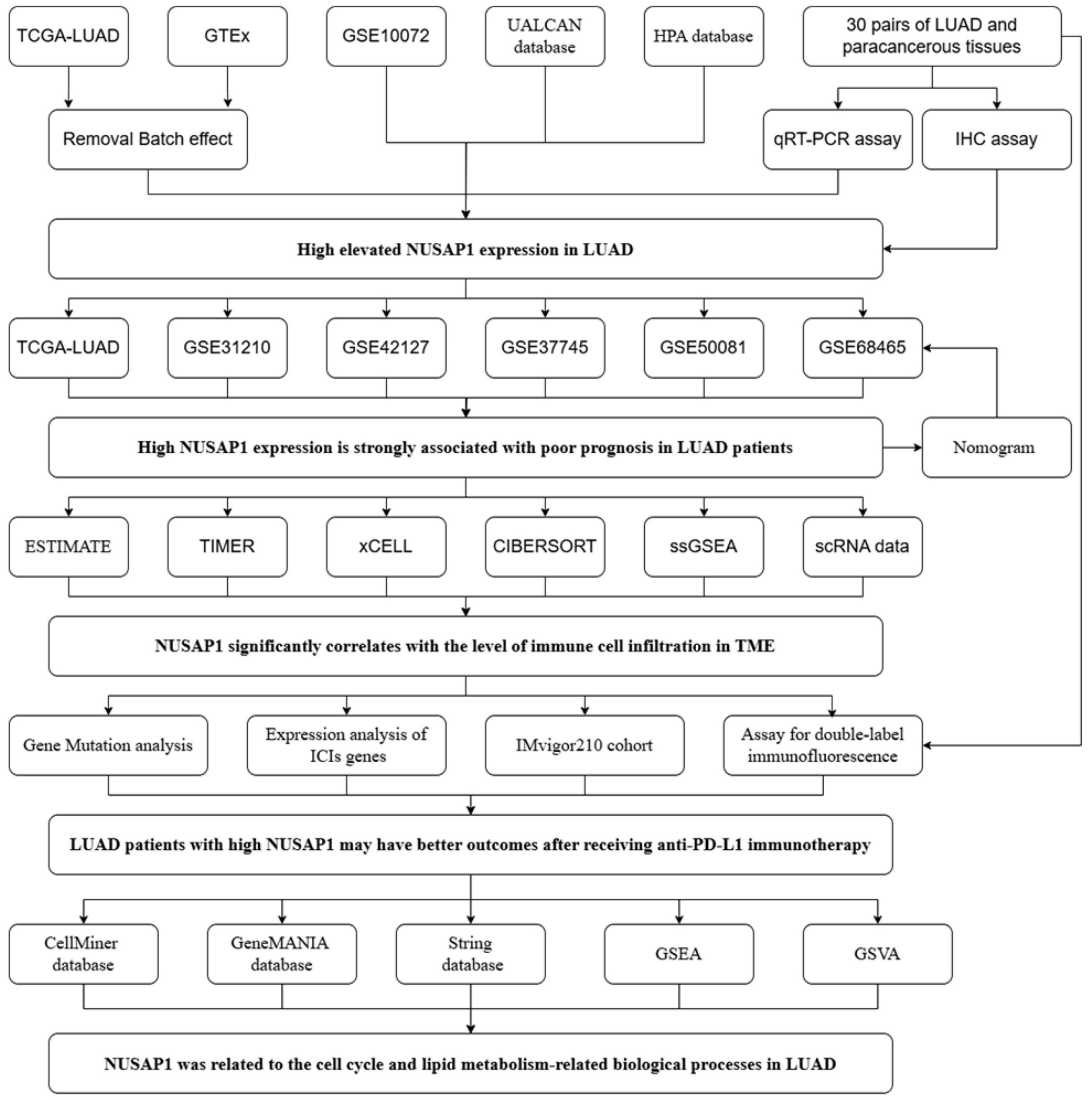
The flow chart.

**Figure 2:**
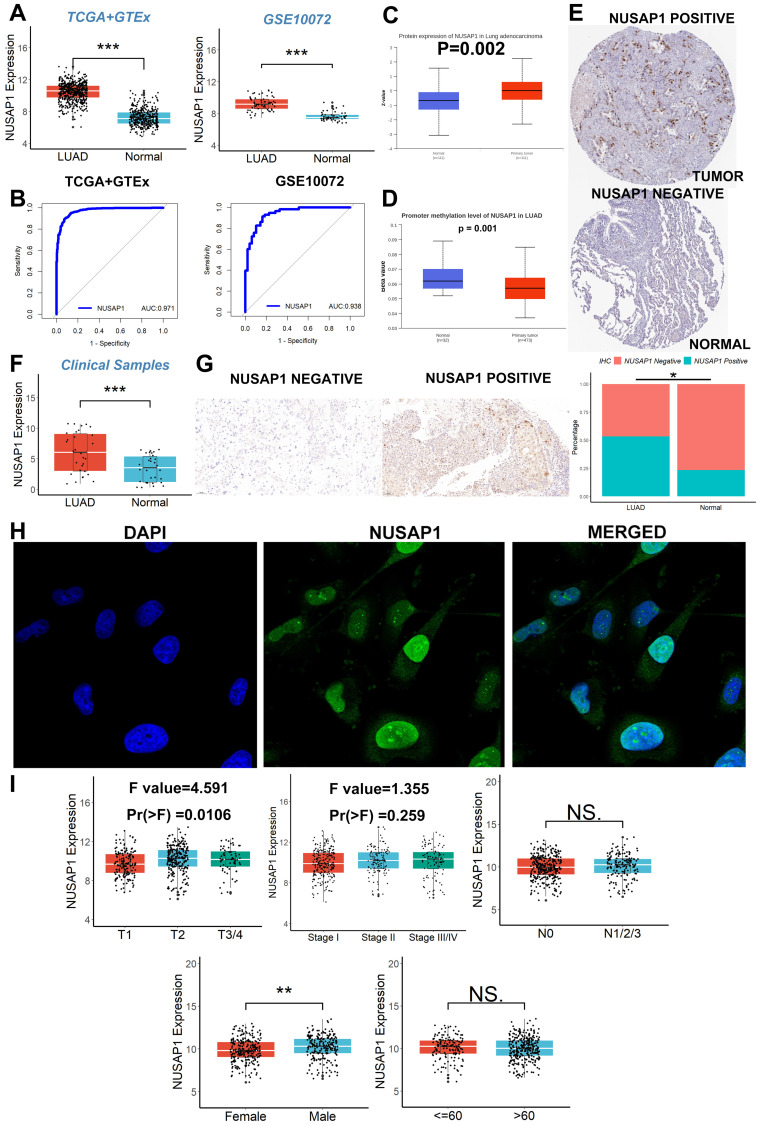
** High elevated NUSAP1 expression in LUAD. (A)** The significant upregulation of NUSAP1 in tumor tissues. **(B)** The remarkable capacity of NUSAP1 to distinguish between tumor and normal samples. **(C)** NUSAP1 expression in the UALCAN database. **(D)** The promoter methylation levels of NUSAP1. **(E)** NUSAP1 expression between tumor and normal samples in the Human Protein Atlas database. **(F)** The result of qRT-PCR. **(G)** The result of IHC assays. **(H)** The result of immunofluorescence staining analysis. **(I)** The relationship between NUSAP1 expression and clinicopathological features. ns, not significant; *p < 0.05; **p <0.01; ***p < 0.001.

The flow chart of this study is shown in Figure 1. We initiated our investigation by examining the expression patterns of NUSAP1 within LUAD across multiple cohorts, including TCGA, GTEx, and GSE10072. Our analysis revealed a significant upregulation of NUSAP1 in tumor tissues (Figure 2A), with a remarkable capacity to distinguish between tumor and normal samples, boasting an area under the curve (AUC) value exceeding 0.930 (Figure 2B). Additionally, leveraging the UALCAN database (http://ualcan.path.uab.edu/index.html), we confirmed a substantial elevation of NUSAP1 expression (Figure 2C) but with lower promoter methylation levels (Figure 2D) in tumor tissues compared to normal tissues. Subsequent exploration utilizing the Human Protein Atlas database (https://www.proteinatlas.org/) underscored a pronounced discrepancy in NUSAP1 expression between tumor and normal samples (Figure 2E). Remarkably, our experimental validations through qRT-PCR (Figure 2F) and IHC assays (Figure 2G) on clinical specimens corroborated the heightened expression of NUSAP1 in tumor tissues. Immunofluorescence staining analysis from the Human Protein Atlas database highlighted the predominant localization of NUSAP1 within the nucleus (Figure 2H). Expanding our inquiry, we delved into the relationship between NUSAP1 expression and clinicopathological features, revealing a significant association with advanced T stage and male gender, while no substantial correlations were observed with N stage, TNM stage, or age among LUAD patients (Figure 2I).

### The potential prognostic power of NUSAP1 in LUAD

**Figure 3:**
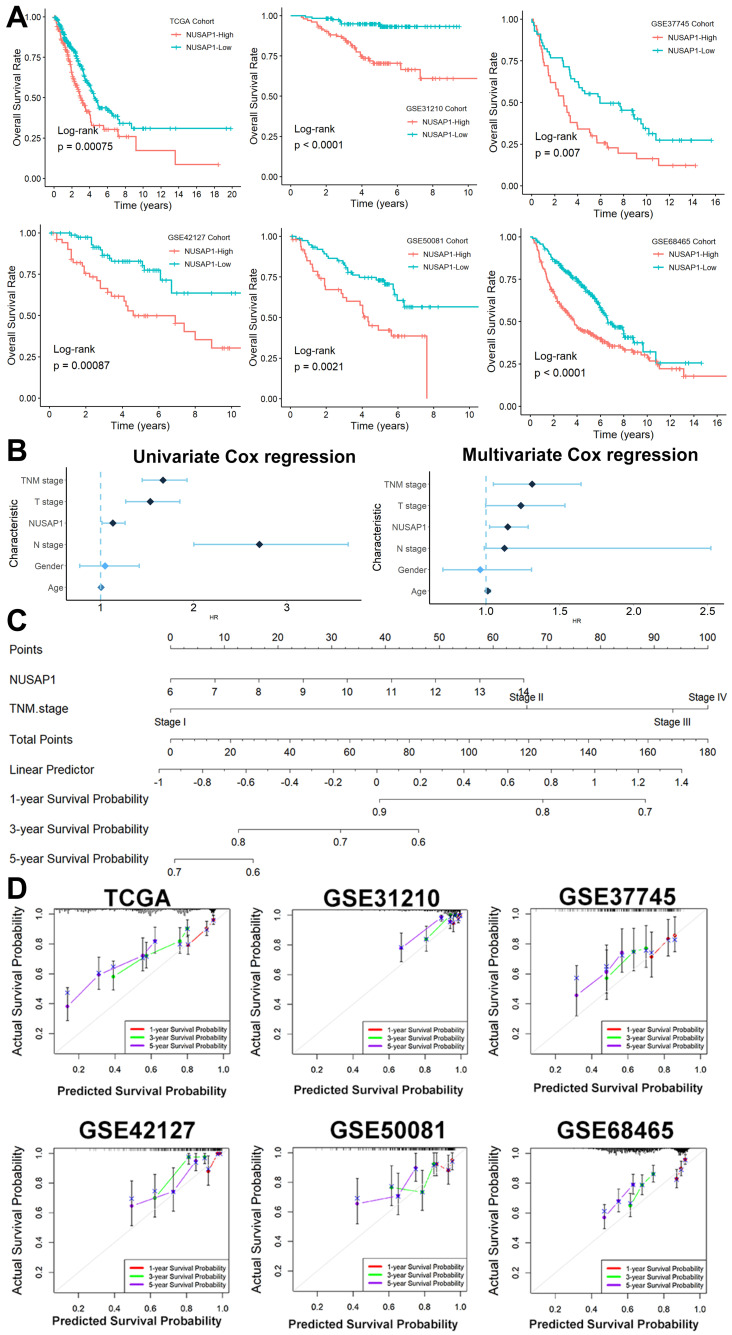
** The potential prognostic power of NUSAP1 in LUAD. (A)** Kaplan-Meier survival analysis across six distinct LUAD cohorts (In TCGA Date, NUSAP1-High=156, NUSAP1-Low=336; In GSE31210 Cohort, NUSAP1-High=102, NUSAP1-Low=124; In GSE37745 Cohort, NUSAP1-High=50, NUSAP1-Low=56; In GSE50081 Cohort, NUSAP1-High=50, NUSAP1-Low=77; In GSE68465 Cohort, NUSAP1-High=225, NUSAP1-Low=214; In GSE42127 Cohort, NUSAP1-High=52, NUSAP1-Low=79). **(B)** The univariate and multivariate Cox regression analyses. **(C)** We developed an overall survival nomogram model capable of predicting 1-, 3-, and 5-year survival probabilities for patients. **(D)** This nomogram model exhibited excellent fitting performance, indicating its reliability in prognostic estimations.

The Kaplan-Meier survival analysis unveiled a robust correlation between elevated NUSAP1 expression and unfavorable survival outcomes across six distinct LUAD cohorts, with a directly proportional relationship between NUSAP1 expression levels and patients' shortened survival duration (Figure 3A). Furthermore, through both univariate and multivariate Cox regression analyses (Figure 3B), we identified NUSAP1 expression along with TNM stage as independent predictors of poor prognosis in LUAD patients. To provide clinicians with a practical tool for prognostic assessment, we developed an overall survival nomogram model capable of predicting 1-, 3-, and 5-year survival probabilities for patients (Figure 3C). Impressively, as illustrated in Figure 3D, this nomogram model exhibited excellent fitting performance, indicating its reliability in prognostic estimations.

### Relationship between NUSAP1 expression and TME

**Figure 4:**
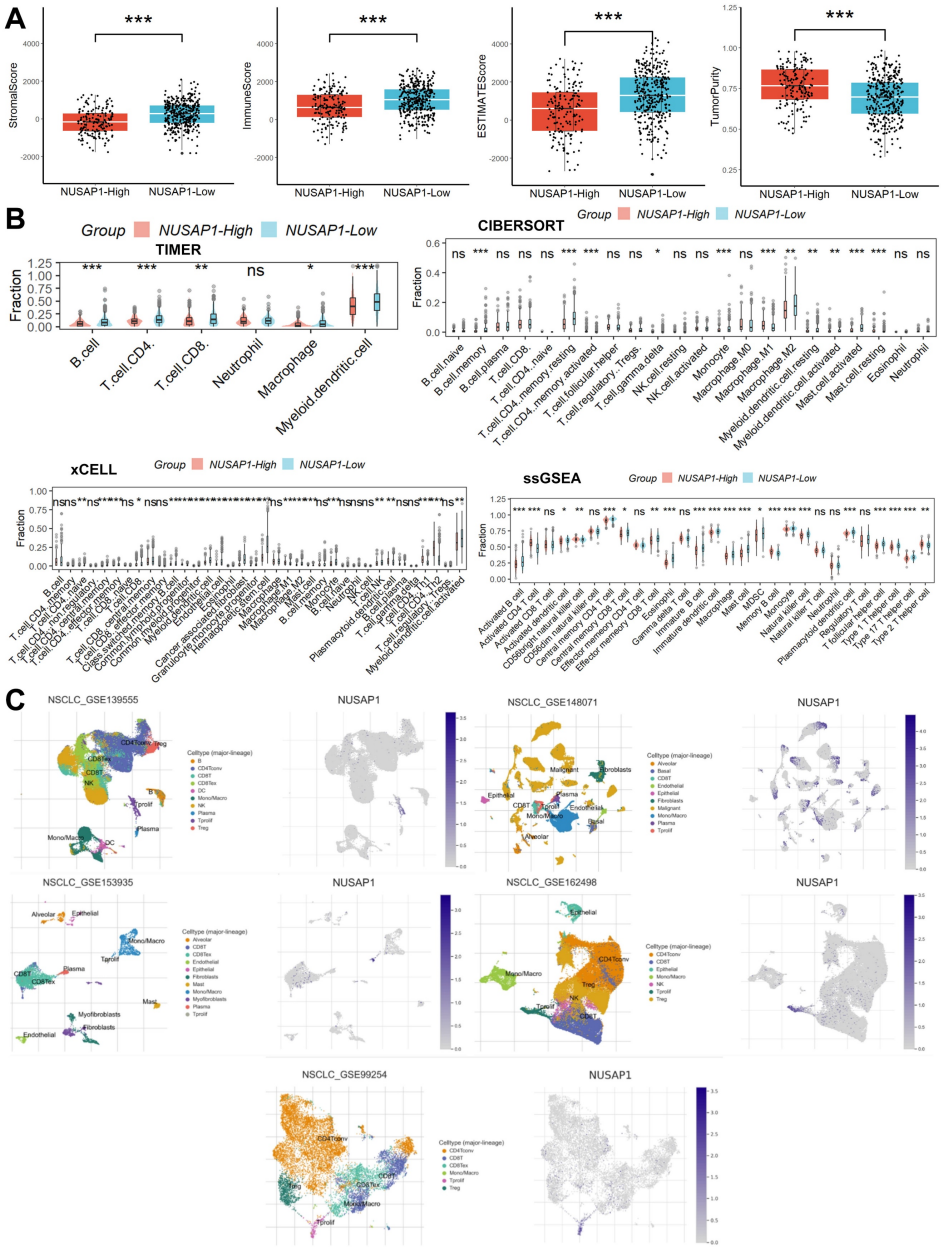
** Relationship between NUSAP1 expression and TME. (A)** Patients with high NUSAP1 expression exhibited a lower proportion of immune and stromal cells but higher tumor cell purity compared to those with low NUSAP1 expression (In TCGA Date, NUSAP1-High=156, NUSAP1-Low=336). **(B)** The reduced proportion of most immune cell types in patients with high NUSAP1 expression compared to those with low NUSAP1 expression. **(C)** The distribution of NUSAP1 expression among Tprolif cells. ns, not significant; *p < 0.05; **p <0.01; ***p < 0.001.

Initially, we evaluated the composition of immune and stromal cells as well as tumor purity in samples from LUAD patients using the ESTIMATE algorithm. This analysis revealed that patients with high NUSAP1 expression exhibited a lower proportion of immune and stromal cells but higher tumor cell purity compared to those with low NUSAP1 expression (Figure 4A). Subsequently, to delve deeper into the distribution of immune cells within the TME, we employed the CIBERSORT, xCELL, TIMER, and ssGSEA algorithms. Remarkably, we observed a reduced proportion of most immune cell types in patients with high NUSAP1 expression compared to those with low NUSAP1 expression (Figure 4B). Lastly, insights gleaned from analyses of five single-cell RNA sequencing datasets unveiled the distribution of NUSAP1 expression among Tprolif cells (Figure 4C).

### Relationship between NUSAP1 expression and immunotherapy efficacy

**Figure 5:**
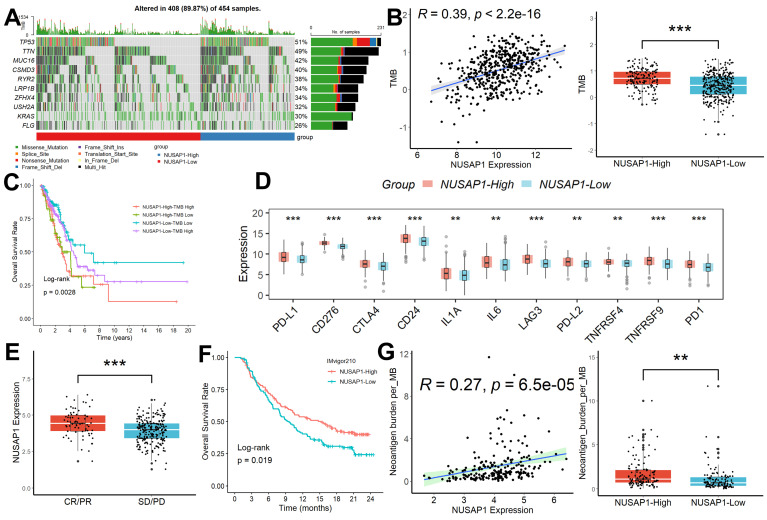
** Relationship between NUSAP1 expression and immunotherapy efficacy. (A)** LUAD samples exhibiting high NUSAP1 expression displayed a higher frequency of somatic mutations compared to those with low NUSAP1 expression. **(B)** NUSAP1 expression exhibited a significant positive correlation with TMB, with LUAD samples featuring high NUSAP1 expression showing markedly elevated TMB values compared to those with low NUSAP1 expression (NUSAP1-High=153, NUSAP1-Low=301). **(C)** Patients with high NUSAP1 expression and elevated TMB values exhibited the poorest survival outcomes (NUSAP1-High-TMB-High =101, NUSAP1-High-TMB-Low=52; NUSAP1-Low-TMB-High =126, NUSAP1-Low-TMB- Low=175). **(D)** The heightened expression of ICIs in LUAD samples with high NUSAP1 expression compared to those with low NUSAP1 expression. **(E)** NUSAP1 expression levels were significantly higher in LUAD samples exhibiting CR/PR to treatment compared to those with SD/PD (CR/PR=68, SD/PD=227). **(F)** Patients with high NUSAP1 expression demonstrated improved survival outcomes relative to those with low NUSAP1 expression (NUSAP1-High=147, NUSAP1-Low=148). **(G)** The significant positive correlation between NUSAP1 expression and neoantigen burden, coupled with higher neoantigen burden values in LUAD samples with high NUSAP1 expression compared to those with low NUSAP1 expression. **p <0.01; ***p < 0.001.

We conducted a comprehensive analysis of mutation data retrieved from LUAD patients using the R package “TCGAmutations,” comparing the mutational landscape between subgroups characterized by different levels of NUSAP1 expression. Notably, LUAD samples exhibiting high NUSAP1 expression (96.3%) displayed a higher frequency of somatic mutations compared to those with low NUSAP1 expression (77.8%) (Figure 5A). Furthermore, NUSAP1 expression exhibited a significant positive correlation with tumor mutation burden (TMB), with LUAD samples featuring high NUSAP1 expression showing markedly elevated TMB values compared to those with low NUSAP1 expression (Figure 5B). Intriguingly, patients with high NUSAP1 expression and elevated TMB values exhibited the poorest survival outcomes (Figure 5C). Additionally, we explored variations in the expression levels of multiple immune checkpoint inhibitors (ICIs) genes across different NUSAP1 expression subgroups. Our analysis revealed heightened expression of ICIs in LUAD samples with high NUSAP1 expression compared to those with low NUSAP1 expression (Figure 5D). Furthermore, we investigated the association between NUSAP1 expression and response to anti-PD-L1 immunotherapy in the IMvigor210 cohort. Remarkably, NUSAP1 expression levels were significantly higher in LUAD samples exhibiting complete response (CR) and partial response (PR) to treatment compared to those with stable disease (SD) and progressive disease (PD) (Figure 5E). Intriguingly, patients with high NUSAP1 expression demonstrated improved survival outcomes relative to those with low NUSAP1 expression (Figure 5F). Ultimately, we observed a significant positive correlation between NUSAP1 expression and neoantigen burden, coupled with higher neoantigen burden values in LUAD samples with high NUSAP1 expression compared to those with low NUSAP1 expression (Figure 5G).

### Prediction of potential tumor-sensitive drugs targeting NUSAP1

**Figure 6:**
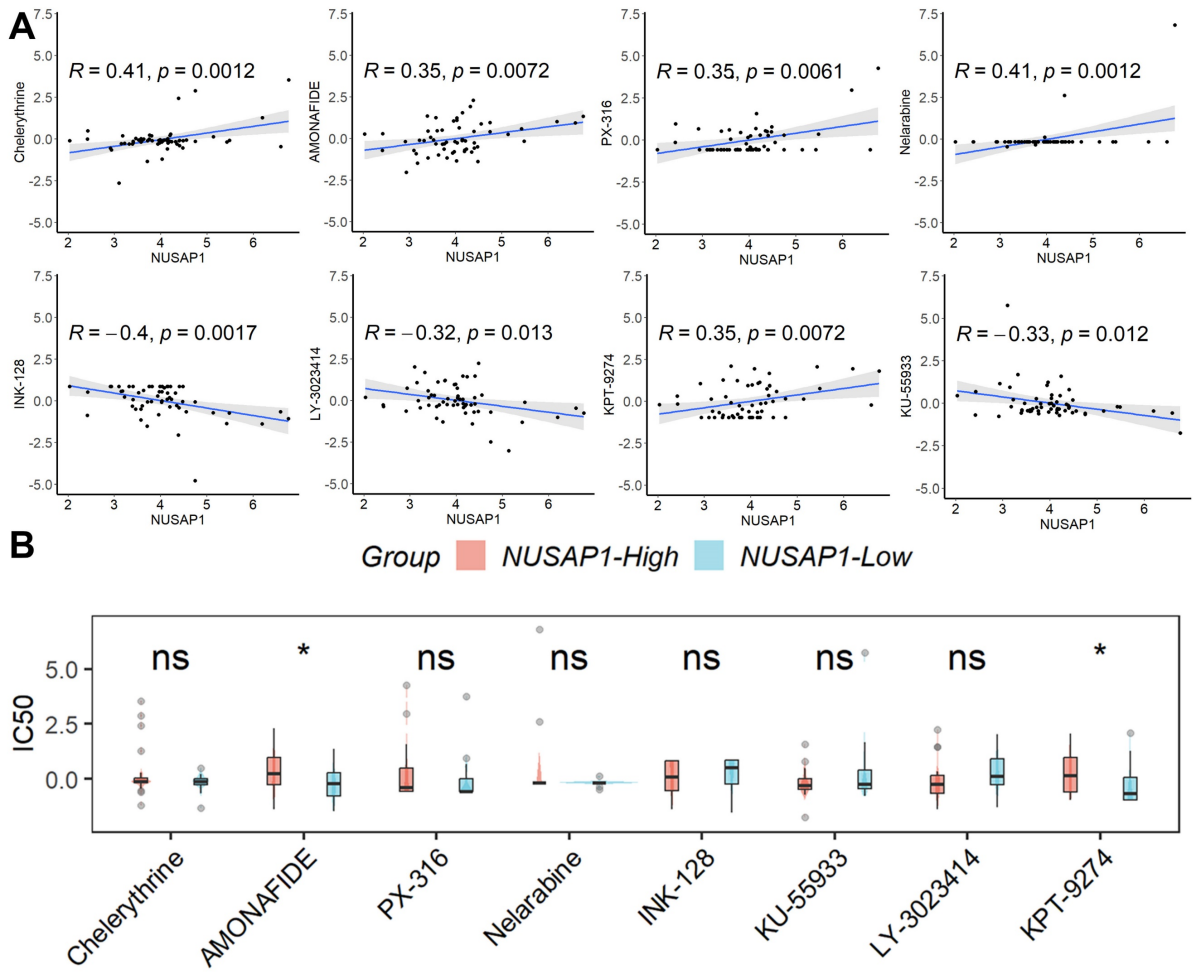
** Prediction of potential tumor-sensitive drugs targeting NUSAP1. (A)** The eight tumor-sensitive drugs. **(B)** Only the IC50 values of AMONAFIDE and KPT-9274 displayed differentiation between samples with high or low NUSAP1 expression. ns, not significant; *p < 0.05.

Based on the findings from CellMiner analysis, we identified eight tumor-sensitive drugs (Figure 6A). Notably, Chelerythrine, AMONAFIDE, PX-316, and Nelarabine exhibited significant positive correlations with NUSAP1 expression, whereas INK-128, KU-55933, LY-3023414, and KPT-9274 showed significant negative correlations with NUSAP1 expression. Of these eight drugs, only the IC50 values of AMONAFIDE and KPT-9274 displayed differentiation between samples with high or low NUSAP1 expression (Figure 6B). These results suggest that amonafide and KPT-9274 may offer heightened efficacy in antitumor therapy for patients with low NUSAP1 expression.

### Relationship between NUSAP1 expression and m6A-related genes

**Figure 7:**
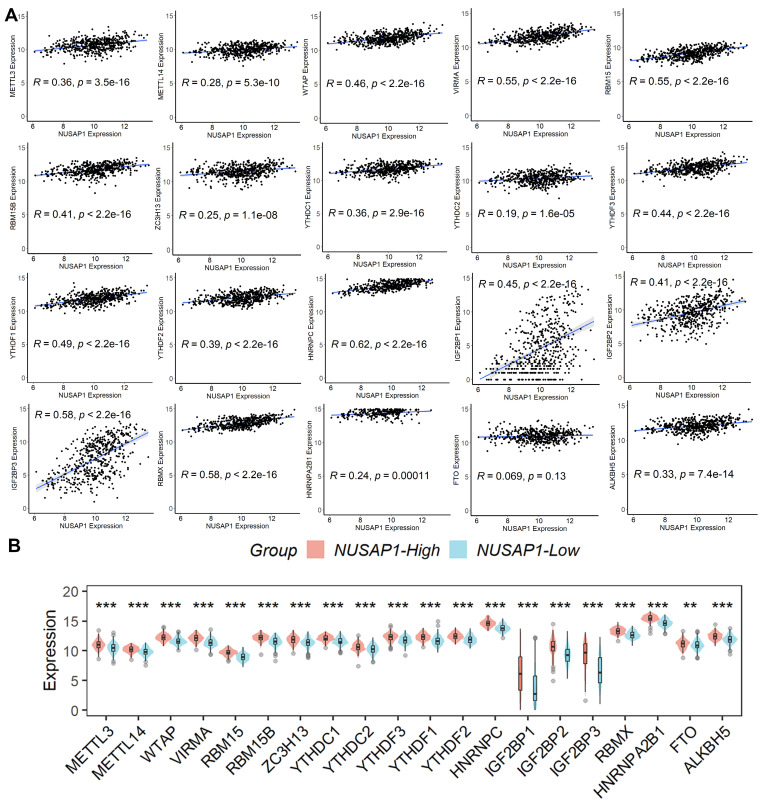
** Relationship between NUSAP1 expression and m6A-related genes. (A)** The significant positive correlation between NUSAP1 expression and the expression of these m6A-related genes (except FTO). **(B)** The notable elevation in the expression of these m6A-related genes in samples. **p <0.01; ***p < 0.001.

Recognizing the pivotal role of m6A modification in LUAD progression 19, we explored the correlation between the expression of various m6A-related genes and NUSAP1 expression. Our analysis revealed a significant positive correlation between NUSAP1 expression and the expression of these m6A-related genes (except FTO) (Figure 7A). Additionally, we observed a notable elevation in the expression of these m6A-related genes in samples with high NUSAP1 expression compared to those with low NUSAP1 expression (Figure 7B). These findings suggest a potential involvement of NUSAP1 in LUAD progression through the mediation of m6A modification pathways.

### Function enrichment analyses

**Figure 8:**
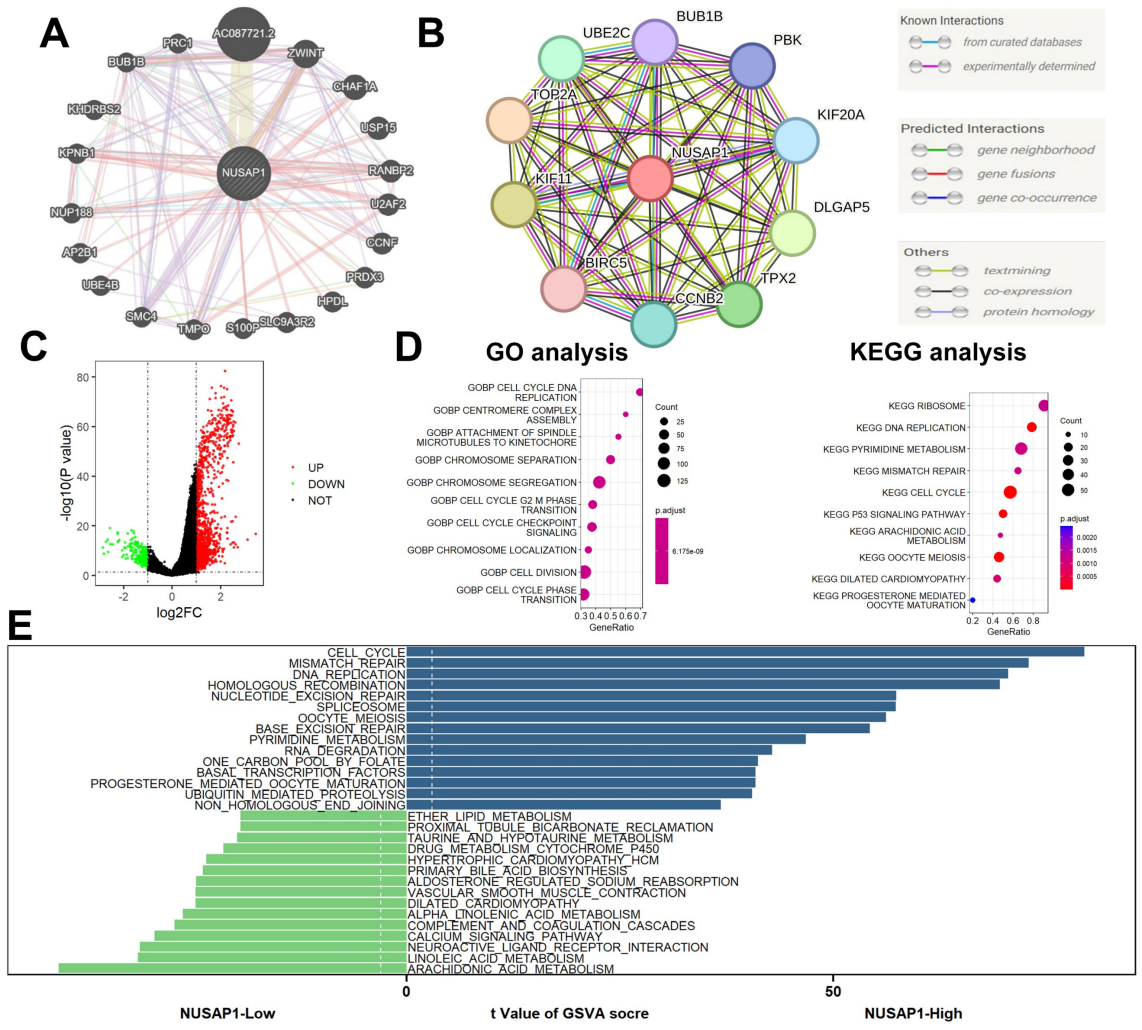
** Function enrichment analyses. (A)** Gene-gene interaction networks. **(B)** Protein-protein interaction networks. **(C)** A total of 1,378 DEGs, comprising 1,145 up-regulated genes and 233 down-regulated genes. **(D)** GO and KEGG analyses. **(E)** GSVA analysis.

We constructed gene-gene interaction networks (Figure 8A) and protein-protein interaction networks (Figure 8B) utilizing the GeneMANIA (http://genemania.org/) 20 and STRING (https://string-db.org/) 21 databases, respectively. Subsequently, differential expression gene (DEG) analysis was conducted between NUSAP1 high- and low-expression samples, employing thresholds of |logFC| > 1 and p-value < 0.05. This analysis revealed a total of 1,378 DEGs, comprising 1,145 up-regulated genes and 233 down-regulated genes (Figure 8C). Furthermore, Gene Ontology (GO) and Kyoto Encyclopedia of Genes and Genomes (KEGG) analyses unveiled that these DEGs were predominantly enriched in cell cycle-related biological processes (Figure 8D). Additionally, Gene Set Variation Analysis (GSVA) highlighted enrichment in cell cycle and lipid metabolism-related biological processes among these DEGs (Figure 8E).

## Discussion

LUAD stands out as one of the most prevalent histological subtypes of non-small cell lung cancer (NSCLC), presenting consistently high morbidity and mortality rates worldwide 22. Despite significant strides in precision medicine, which have unveiled a spectrum of biomarkers and facilitated the adoption of personalized therapeutic approaches, challenges persist in targeted therapies and immunotherapies for LUAD patients 23, 24. As such, LUAD remains a significant global public health concern. Thus, the quest for biomarkers capable of accurately prognosticating outcomes and guiding immunotherapeutic efficacy holds paramount importance in shaping clinical interventions for LUAD patients. In our study, we established a significant correlation between elevated NUSAP1 expression and unfavorable prognosis in LUAD using multi-omics analysis. Additionally, we investigated disparities in the TME, mutation profiles, and response to immunotherapy across LUAD samples with varying NUSAP1 expression levels. This comprehensive analysis not only enhances comprehension of LUAD tumor immunology but also offers valuable insights for refining therapeutic strategies and ultimately improving LUAD patient outcomes.

Multiple immune cell populations within the TME play pivotal roles in the onset, progression, and treatment outcomes of LUAD patients 25. A comprehensive understanding of TME characteristics is paramount for devising novel immunotherapeutic strategies. Tumor-associated macrophages (M2-type) and IL-10-secreting B cells exhibit pro-tumorigenic traits and immunosuppressive functions 26, 27. Regulatory T cells facilitate tumor growth by suppressing anti-tumor responses 28. CD8+ T cells induce direct cytotoxic effects on cancer cells through the secretion of granzymes and perforins 29. Dendritic cells capture tumor antigens, instigating CD8+ or CD4+ T cell-mediated immune responses 30. Conversely, tumor-associated macrophages (M1-type) secrete tumor necrosis factor-alpha and nitric oxide to eliminate tumor cells and bolster T cell-mediated immune responses 31. In this study, we utilized various algorithms, including ESTIMATE, CIBERSORT, xCELL, TIMER, and ssGSEA, to assess the TME of LUAD samples. Our findings revealed a robust correlation between NUSAP1 expression and immune cell infiltration. Notably, we observed diverse NUSAP1 expression patterns, with high NUSAP1 expression associated with reduced immune cell infiltration compared to samples exhibiting low NUSAP1 expression. Subsequent analysis of multiple single-cell RNA sequencing datasets unveiled predominant NUSAP1 expression on Tprolif cells, supported by gene enrichment analyses. GSEA and GSVA results highlighted the association between NUSAP1 and lipid metabolism, a process pivotal in cancer cell tumorigenesis, disease progression, immune cell recruitment, and modulation of the immune microenvironment response 32. Importantly, lipid metabolism may impact the biological activity of Tprolif cells 33.

The TME harboring multiple immunotherapy-associated targets such as PDCD1, PD-L1, LAG3, and CTLA4 can significantly influence the efficacy of immunotherapy in LUAD patients 34. Consequently, immunotherapeutic approaches targeting the TME to impede tumor growth have garnered considerable attention in recent years. However, resistance to immunotherapy in LUAD stems from the intricate nature of the immune system. One of the foremost unresolved challenges in immunotherapy research is the absence of specific biomarkers to accurately predict the response to immunotherapy in LUAD 35. In our study, we observed that LUAD samples exhibiting high NUSAP1 expression displayed increased somatic mutation frequency, elevated TMB values, and heightened expression of ICI genes compared to samples with low NUSAP1 expression. Furthermore, analysis of the IMvigor210 cohort revealed that patients with high NUSAP1 expression exhibited superior survival rates and treatment response rates in comparison to those with low NUSAP1 expression. These findings suggest that NUSAP1 holds promise as a predictive biomarker for the efficacy of anti-PD-L1 therapy in LUAD patients. Consequently, our study advocates for the inclusion of LUAD patients with high NUSAP1 expression in future multicenter trials investigating anti-PD-L1 immunotherapy. Furthermore, given that m6A-modified genes often exhibit oncogenic functions in cancer, m6A-related therapies, such as modulation or inhibition of m6A modifications, hold potential as promising cancer treatments 36. In this study, we identified a positive correlation between m6A modification genes and NUSAP1 expression. Notably, there was a significant increase in the expression of these m6A modifier genes in LUAD samples with high NUSAP1 expression compared to those with low NUSAP1 expression. These findings suggest that NUSAP1 could play a role in predicting the efficacy of m6A-related therapies for managing LUAD.

In our study, utilizing the CellMiner database, we discovered that amonafide and KPT-9274 show increased effectiveness in anti-tumor therapy for patients exhibiting low NUSAP1 expression. Amonafide, currently under clinical development for cancer treatment, acts as a DNA intercalator, which contains a 5-position amine that undergoes variable acetylation to produce a toxic metabolite in humans, leading to heightened adverse effects and complicating the dosing of amonafide 37. Amonafide demonstrates significant inhibition of tumor growth, reduction in tumor size, and promising activity in treating advanced breast cancer 38 and acting as a second-line option for acute myeloid leukemia 39. Unfortunately, in a Phase II clinical study, 18 recruited NSCLC patients were unable to benefit from Amonafide treatment 40. Additionally, KPT-9274, a dual-specific inhibitor targeting PAK4 and nicotinamide phosphoribosyltransferase, effectively suppressed the growth, survival, and migration of triple-negative breast cancer 41, 42, kidney cancer 43, acute myeloid leukemia 44, and ovarian cancer cells 45. It could also increase the therapeutic sensitivity of pancreatic neuroendocrine tumors to everolimus 46. Regrettably, there is currently no study reporting whether treatment with KPT-9274 benefits LUAD patients. This necessitates the recruitment of a specific number of patients for clinical drug trials in the future to validate its efficacy.

## Conclusion

In conclusion, the present study identified NUSAP1 as a potential prognostic and immunotherapeutic biomarker for LUAD patients, which warrants further investigation.
